# Rapid, scalable assay of amylin-β amyloid co-aggregation in brain tissue and blood

**DOI:** 10.1016/j.jbc.2023.104682

**Published:** 2023-04-06

**Authors:** Deepak Kotiya, Noah Leibold, Nirmal Verma, Gregory A. Jicha, Larry B. Goldstein, Florin Despa

**Affiliations:** 1 Department of Pharmacology and Nutritional Sciences, University of Kentucky, Lexington, Kentucky, USA; 2 The Research Center for Healthy Metabolism, University of Kentucky, Lexington, Kentucky, USA; 3 Sanders-Brown Center on Aging, University of Kentucky, Lexington, Kentucky, USA; 4 Department of Neurology, University of Kentucky, Lexington, Kentucky, USA

**Keywords:** amyloid, Alzheimer’s disease, type 2 diabetes, enzyme-linked immunoassay, amylin

## Abstract

Islet amyloid polypeptide (amylin) secreted from the pancreas crosses from the blood to the brain parenchyma and forms cerebral mixed amylin-β amyloid (Aβ) plaques in persons with Alzheimer’s disease (AD). Cerebral amylin-Aβ plaques are found in both sporadic and early-onset familial AD; however, the role of amylin-Aβ co-aggregation in potential mechanisms underlying this association remains unknown, in part due to lack of assays for detection of these complexes. Here, we report the development of an ELISA to detect amylin-Aβ hetero-oligomers in brain tissue and blood. The amylin-Aβ ELISA relies on a monoclonal anti-Aβ mid-domain antibody (detection) and a polyclonal anti-amylin antibody (capture) designed to recognize an epitope that is distinct from the high affinity amylin-Aβ binding sites. The utility of this assay is supported by the analysis of molecular amylin-Aβ codeposition in postmortem brain tissue obtained from persons with and without AD pathology. By using transgenic AD-model rats, we show that this new assay can detect circulating amylin-Aβ hetero-oligomers in the blood and is sensitive to their dissociation to monomers. This is important because therapeutic strategies to block amylin-Aβ co-aggregation could reduce or delay the development and progression of AD.

Alzheimer’s disease (AD) is associated with dysregulated pancreatic hormones such as insulin leading to brain insulin resistance that can exacerbate AD pathology (1, 2). Amylin, a pancreatic β-cell hormone cosynthesized and cosecreted with insulin (3), crosses from the blood to the brain parenchyma (4) and is involved in the central regulation of satiation (5). In persons with type 2 diabetes mellitus, amylin forms pancreatic amyloid (>95% prevalence at autopsy) which contributes to type 2 diabetes pathogenesis by inducing β-cell apoptosis and β-cell mass depletion (6, 7, 8). Amylin also synergistically co-aggregates with vascular and brain parenchymal β amyloid (Aβ) in the brain in both sporadic Alzheimer’s disease (sAD) and early onset familial Alzheimer’s disease (fAD) (9, 10, 11, 12, 13). Using APPswe/PS1dE9 (APP/PS1) rats expressing human amylin specifically in the pancreas (murine amylin is nonamyloidogenic), chronic exposure to circulating amyloid-forming amylin was found to promote cerebrovascular and parenchymal amylin-Aβ deposition (12, 13), consistent with findings in human AD brains (9, 10, 11, 12, 13). These results (9, 10, 11, 12, 13) suggest an association between pancreatic amyloid-forming amylin and Aβ pathology. Whether co-aggregation of amylin and Aβ is the mechanism underlying this association remains unknown.

The amino acid sequences of amylin and Aβ peptides promoting amylin-Aβ co-aggregation (*i.e.*, formation of amylin-Aβ hetero-oligomers) were assessed by using synthetic amylin and Aβ peptides (14, 15, 16, 17, 18, 19, 20). Heterologous seeding between amylin and various Aβ fragments (*i.e.*, Aβ_42_ and Aβ_40_) propagates amyloid formation comparable to that of homogenous amylin amyloid (14, 15, 16). Co-expression of the two peptides in cells promotes *in vivo* amylin-Aβ hetero-amyloid formation and cytotoxicity (20, 21). Immunoprecipitated amylin from human AD brain tissue analyzed by Western blot using an anti-Aβ antibody detected Aβ immunoreactivity indicates that the amylin and Aβ peptides aggregate in the human AD brain forming amylin-Aβ hetero-oligomers that are SDS-soluble (9). These data (9, 14, 15, 16, 17, 18, 19, 20) collectively confirm that amylin-Aβ hetero-oligomers can be detected and quantified and may serve as a marker of amylin-Aβ established interaction and its correlation with AD pathology.

Although the need to quantify molecular amylin-Aβ interaction in human AD has been recognized (9, 10, 11, 12, 13), the complexity and lack of scalability of traditional methods (such as immunoprecipitation, Western blot, circular dichroism, and electron microscopy) have hampered studies requiring larger sample sizes. For example, studies of amylin-Aβ hetero-oligomers in human brain homogenates by coimmunoprecipitation and Western blot were conducted in only a few samples (9). Furthermore, denaturing conditions present in SDS gels may alter the structural stability and size of the amylin-Aβ hetero-oligomer, unlike other methods such as ELISA in which proteins remain in their native state. The levels of amylin-Aβ hetero-oligomers in the brains of humans with AD compared to those in the brains of unaffected persons remain unknown. Here, we report the development of an ELISA to detect amylin-Aβ hetero-oligomers in brain tissue and blood. Using the new assay, we screened human temporal cortex homogenates for amylin-Aβ hetero-oligomers. Transgenic AD-model rats were used to detect circulating amylin-Aβ hetero-oligomers in the blood. Assessing directly co-aggregated amylin-Aβ in brain tissues and blood may help better delineate the mechanisms underlying the association between pancreatic amyloid-forming amylin and cerebral Aβ pathology. This is important because therapeutic strategies to block amylin-Aβ co-aggregation could reduce or delay the development and progression of AD.

## Results

### A specific ELISA for measuring amylin-Aβ hetero-oligomers in human AD brains

**Figure 1:**
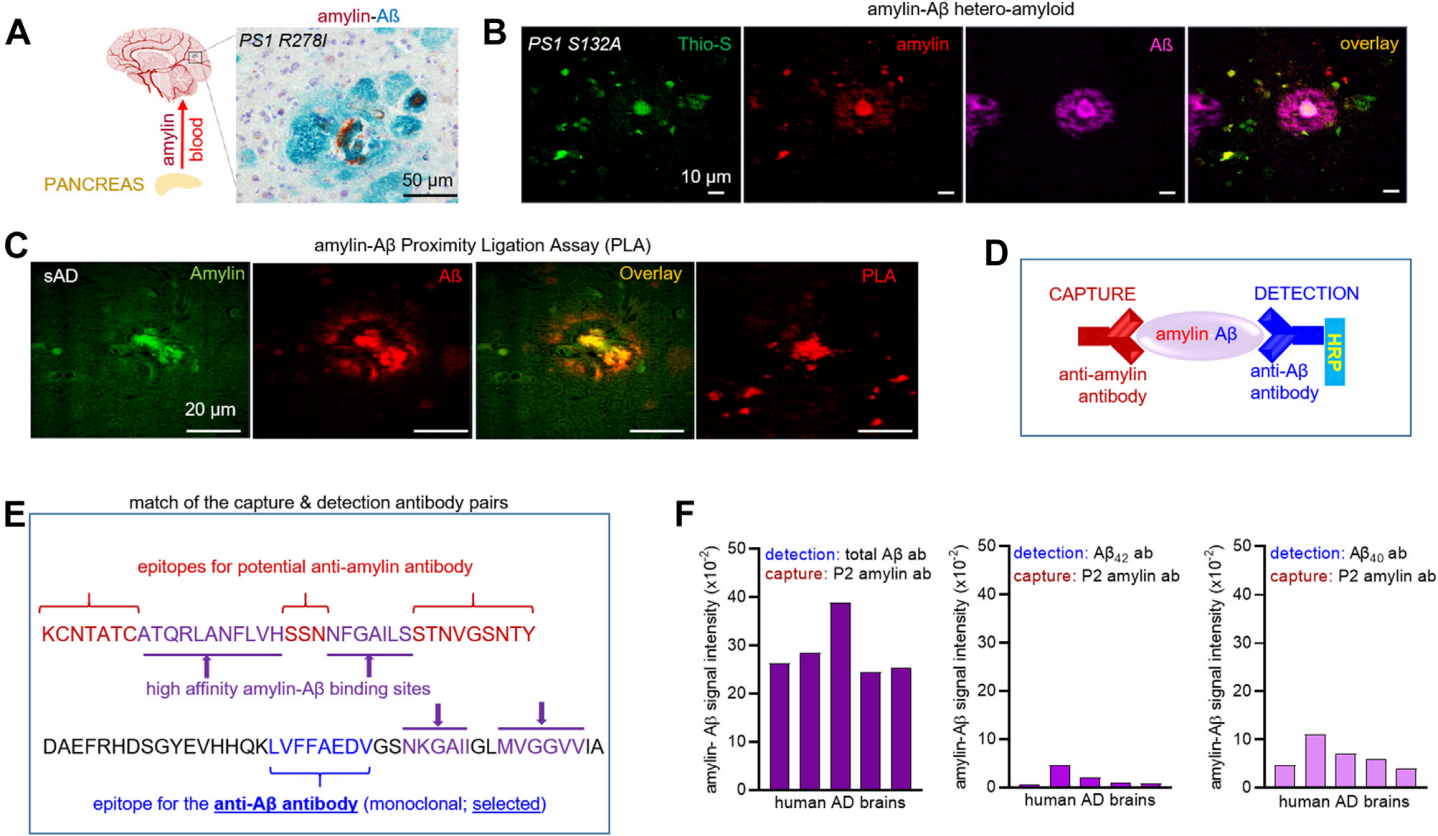
**Detection of amylin-Aβ hetero-oligomers in human AD brain tissues.***A*, the schematic illustration of amylin-Aβ hetero-amyloid formation in the brain along with an example of cerebral amylin-Aβ codeposition reported previously (12). The section through the brain of a PS1 mutation carrier shows cerebral Aβ deposits (*green*) and amylin (*brown*) forming the plaque core and intercalated amylin-Aβ deposits. *B*, confocal microscopic analysis of a section through the brain of PS1 mutation carrier stained with Thioflavin S (Thio S; *green*), amylin (*red*), and Aβ (*magenta*). *C*, confocal microscopic analysis of a section through the brain of a person with sAD stained with amylin (*green*) and Aβ (*red*) and further subjected to an amylin-Aβ PLA (*red*). *D*, schematic representation of an amylin-Aβ sandwich ELISA with the anti-amylin antibody used as the capture antibody and anti-Aβ antibody conjugated with horseradish peroxidase (HRP) used as the detection antibody. *E*, the amino acid sequence analysis of amylin and Aβ peptides indicating the epitope regions against the anti-amylin capture and anti-Aβ detection antibodies used in the amylin-Aβ sandwich ELISA. *F*, amylin-Aβ immunoreactivity signal intensity measured in human sAD brain tissue homogenates (n = 5) using the P2 amylin antibody as the capture antibody, and total Aβ or Aβ_42_ or Aβ_40_ antibodies as the detection antibody in the amylin-Aβ sandwich ELISA. Scale bars represent 10 μm in (*B*) and 20 μm in (*C*). Aβ, β amyloid; AD, Alzheimer’s disease; PLA, proximity ligation assay; PS1, presenilin 1; sAD, sporadic Alzheimer’s disease.

An example of cerebral amylin-Aβ codeposition reported previously (12) is shown in Figure 1*A*. The section through the brain of a PS1 mutation carrier shows cerebral Aβ deposits (green) and amylin (brown) forming the plaque core and intercalated amylin-Aβ deposits. Amylin-Aβ codeposits have biochemical characteristics of amyloid, as demonstrated by thioflavin S (Thio-S) costaining (Fig. 1*B*) and involve direct molecular amylin-Aβ binding, as suggested by the proximity ligation assay (PLA) (Fig. 1*C*). To assess molecular amylin-Aβ codeposition in human AD brains quantitatively, we developed an amylin-Aβ sandwich ELISA that relies on an anti-Aβ antibody (detection) and an anti-amylin antibody (capture) (Fig. 1*D*).

For co-aggregated amylin-Aβ (amylin-Aβ hetero-oligomers) to be detected by a sandwich ELISA, the molecular assembly must contain exposed epitopes that are distinct from amylin-Aβ binding sites on both amylin and Aβ peptides (Fig. 1*E*). These epitopes were identified based on the analyses of amino acid sequences of amylin and Aβ peptides indicating regions on the amylin and Aβ peptides that promote amylin-Aβ oligomerization (14, 16). For performing amylin-Aβ sandwich ELISA, three well-characterized monoclonal anti-Aβ antibodies were tested as potential detection antibodies, including those that recognize the 17 to 24 amino acids of Aβ (total Aβ antibody), the Aβ isoform ending at the 42nd amino acid (Aβ_42_ antibody), and the isoform ending at 40th amino acid (Aβ_40_ antibody) (22, 23, 24). For capturing antibodies, we tested both amylin C- and N-termini antibodies. Based on the immunoreactivity signal intensity measured in the solutions of amylin-Aβ hetero-oligomers (Fig. S1), a polyclonal anti-amylin antibody (P2) designed to recognize an N-terminus amylin epitope was selected as the capture antibody. The P2 capture antibody matched with the antitotal Aβ detection antibody showed a robust amylin-Aβ immunoreactivity signal intensity in human AD brain tissue homogenates (Fig. 1*F*). Therefore, our specific amylin-Aβ sandwich ELISA for measuring amylin-Aβ co-aggregation in AD brain tissue homogenates relies on a monoclonal anti-Aβ mid-domain antibody (detection) and a polyclonal anti-amylin antibody (capture). This assay will recognize only amylin-Aβ molecular assemblies that contain at least one exposed Aβ within amylin-positive molecular aggregates.

### Specificity of sandwich ELISA to detection of prefibrillar amylin-Aβ hetero-oligomers

**Figure 2:**
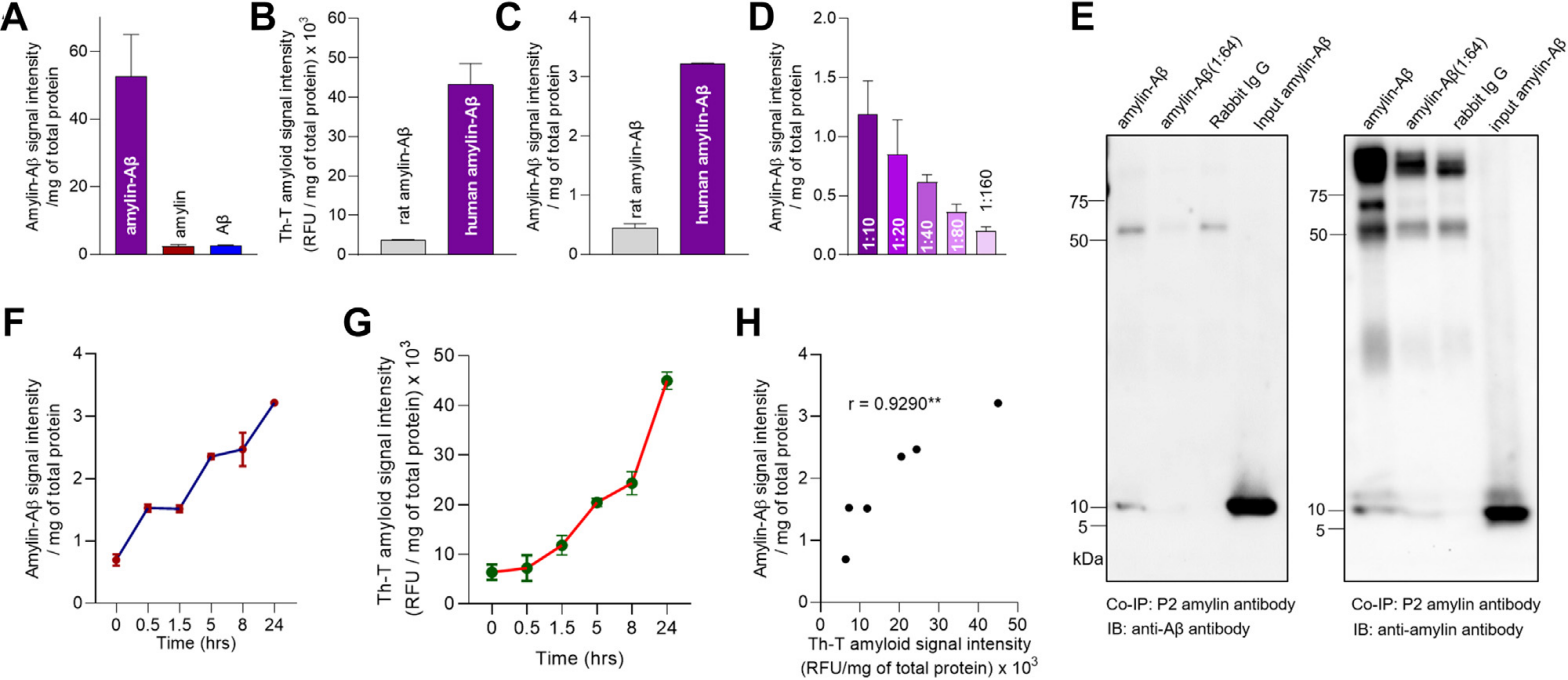
**Specificity and sensitivity of the amylin-Aβ sandwich ELISA to detection of amylin-Aβ hetero-oligomers.***A*, the average amylin-Aβ immunoreactivity signal intensities measured in amylin-Aβ (30 μM amylin and 16.2 μM Aβ; mixed in a 1:1 ratio to give final concentrations of 15 μM amylin and 8.1 μM Aβ), amylin (30 μM), and Aβ (16.2 μM) aggregates using the amylin-Aβ sandwich ELISA. *B*, thioflavin T (Th-T) fluorescence signal intensities measured in the solutions of human amylin-Aβ and rat amylin-Aβ aggregates (same peptide concentrations as in *A*). *C*, amylin-Aβ immunoreactivity signal intensities measured by the amylin-Aβ sandwich ELISA in rat amylin-Aβ and human amylin-Aβ hetero-oligomers. *D*, amylin-Aβ immunoreactivity signal intensities measured by the amylin-Aβ sandwich ELISA in amylin-Aβ dilutions (1:10–1:160) using the same aliquots of amylin-Aβ as in (*B*). *E*, coimmunoprecipitation of amylin using the P2 amylin antibody in amylin-Aβ aggregates (undiluted and 1:64 diluted) followed by Western blot analysis of Aβ and amylin in amylin IP eluates. *F*, amylin-Aβ immunoreactivity signal intensities measured by the amylin-Aβ sandwich ELISA in the fraction collected at various time points of human amylin (30 μM) incubated with human Aβ_40_ (16.2 μM). *G*, Th-T fluorescence signal intensities measured in the same fraction collected as in (*F*). *H*, pairwise correlation analyses of amylin-Aβ signal intensity measured in (*F*) *versus* Th-T fluorescence signal intensities measured in (*G*). Data are mean ± SD. Data are presented as correlation analysis, Pearson’s correlation ∗∗*p* < 0.01 in (*H*). Aβ, β amyloid.

Specificity of amylin-Aβ sandwich ELISA was confirmed using a solution phase amylin-Aβ aggregation assay described in (14). Synthetic human amylin and Aβ_40_ peptides were incubated for promoting the amylin-Aβ aggregation and subjected to the amylin-Aβ sandwich ELISA. Protein solutions created by the incubation of similar concentrations of amylin or Aβ_40_ alone, as used in preparing amylin-Aβ aggregates, provided negative controls for the detection of amylin-Aβ immunoreactivity. A robust amylin-Aβ immunoreactivity signal was detected in co-aggregated amylin-Aβ solution, but not in the homogenous amylin and Aβ solutions (Fig. 2*A*). The aggregated amylin-Aβ solution was assayed for biochemical characteristics of amyloid by measuring the thioflavin-T (Th-T) fluorescence signal intensity. Because rat amylin is nonamyloidogenic (25), negative controls for the amylin-Aβ amyloid characteristics were protein solutions created by incubation of equal concentrations of Aβ and rat amylin. Both Th-T fluorescence and amylin-Aβ ELISA signal intensities were greatly reduced in rat amylin-Aβ solutions compared to that in human amylin-Aβ solutions (Fig. 2, *B* and *C*). The average amylin-Aβ ELISA signal intensity in human amylin-Aβ co-aggregation assays is ∼7-fold higher than that corresponding to rat amylin-Aβ co-aggregation (Fig. 2*C*). The propensity of aggregate formation by rat amylin with Aβ is much lower than aggregate formation by human amylin with Aβ, consistent with previously published data (20).

From the same aliquot as in Figure 2*A*, serial dilutions of amylin-Aβ hetero-oligomers were assayed using the amylin-Aβ sandwich ELISA (Fig. 2*D*) and the limit of detection (LOD) was estimated. The LOD of the amylin-Aβ sandwich ELISA is 0.02 ng/mg total protein. Next, we used the same anti-amylin and anti-Aβ antibodies as in the sandwich ELISA (*i.e.*, P2 amylin antibody and horseradish peroxidase (HRP)-conjugated anti-Aβ antibody) for coimmunoprecipitation of amylin in amylin-Aβ aggregates (undiluted and 1:64 diluted) followed by a direct Western blot analysis of Aβ in amylin immunoprecipitation (IP) eluates. Low molecular weight (∼10 kDa) hetero-oligomers (likely amylin-Aβ dimers) were present within the hetero-oligomeric solution (Fig. 2*E*). Following further dilution (1:64) of the input aliquot, low molecular weight (∼10 kDa) hetero-oligomers are faintly shown on Western blot, consistent with the lower sandwich ELISA signal intensity show in Figure 2*D*.

To test whether prefibrillar amylin-Aβ oligomers can be detected *via* the sandwich ELISA method, the time evolution of the sandwich ELISA signal intensity in an incubated solution of amylin and Aβ (Fig. 2*F*) was analyzed in parallel for amyloid fibril formation *via* measurements of Th-T fluorescence (Fig. 2*G*). The measurements were conducted at six different time points (0, 0.5, 1.5, 5, 8, and 24-h) postincubation. The results show that increased Th-T fluorescence signal intensities are associated with greater sandwich ELISA signal intensities (*r* = 0.93; *p* < 0.01) and a 3-fold increase from the baseline (Fig. 2*H*). Our interpretation is that amylin-Aβ ELISA can detect prefibrillar amylin-Aβ hetero-oligomers.

### Sensitivity of amylin-Aβ sandwich ELISA to dissociation of amylin-Aβ hetero-oligomers

**Figure 3:**
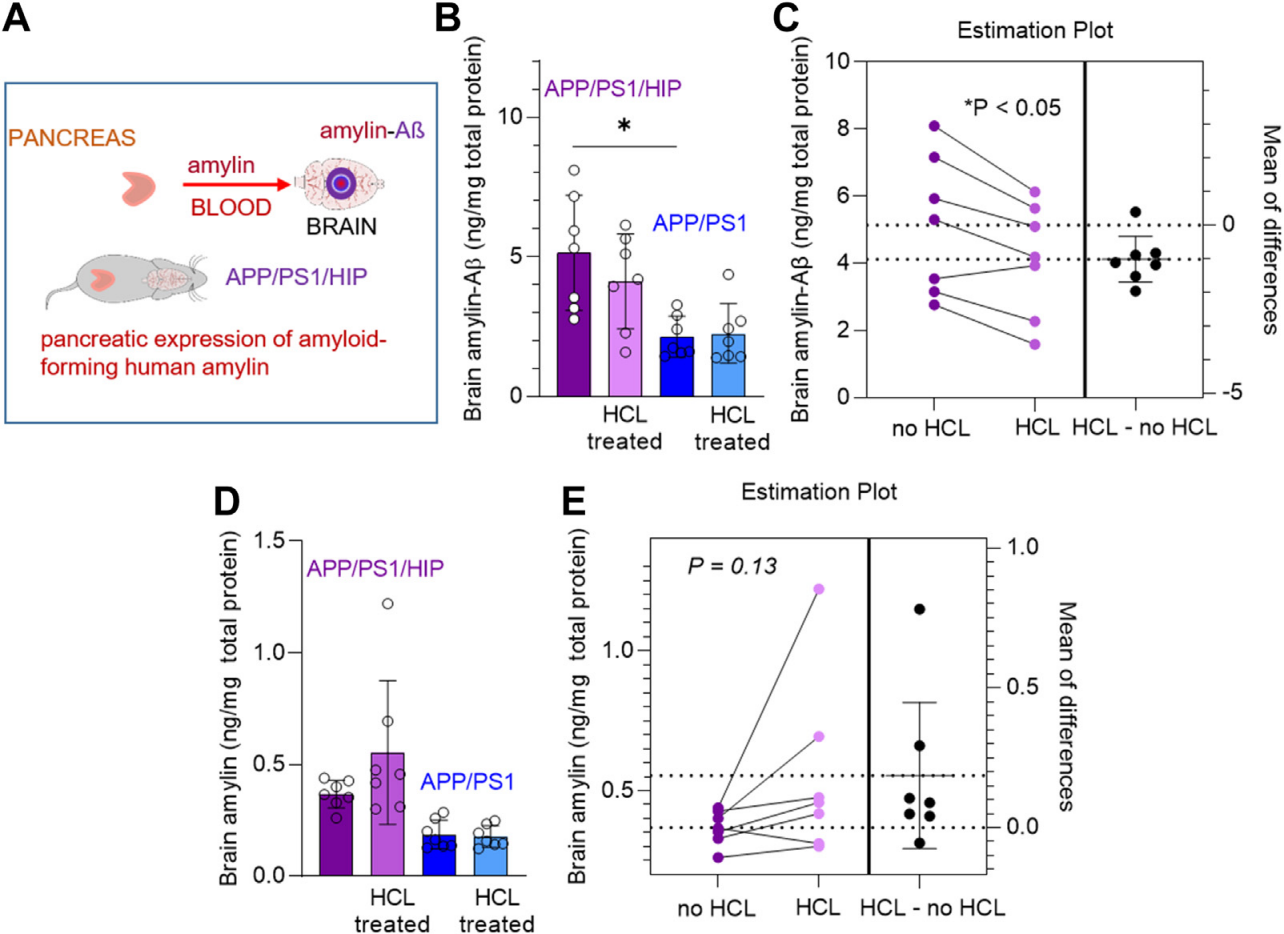
**Sensitivity of amylin-Aβ sandwich ELISA to dissociation of amylin-Aβ hetero-oligomers.***A*, schematic illustration describing APP/PS1/HIP rats expressing human amylin in the pancreas. *B*, amylin-Aβ concentrations measured using the amylin-Aβ sandwich ELISA in brain homogenates from age-matched APP/PS1 and APP/PS1/HIP rats (age, 16 months, n = 7 males/group) with and without hydrochloric acid (HCl) treatment to fragment the amylin-Aβ hetero-oligomers. *C*, pairwise comparison for the level of amylin-Aβ concentrations measured in (*B*) with and without HCl treatment for APP/PS1/HIP brain homogenates. *D*, amylin concentrations measured in same samples as in (*B*) using indirect amylin ELISA. *E*, pairwise comparison of the levels of amylin concentrations measured in (*D*) with and without HCl treatment for APP/PS1/HIP brain homogenates. Data are mean ± SD. Kruskal–Wallis one-way of variance test in (*B*) *∗p* < 0.05 and (*D*). Pairwise comparisons and estimation plots, two-tailed, paired *t* test ∗*p* < 0.05 in (*C*) and (*E*). Aβ, β amyloid; APP, amyloid precursor protein; HIP, human islet amyloid polypeptide; PS1, presenilin 1.

To further confirm that the amylin-Aβ sandwich ELISA is sensitive to amylin-Aβ hetero-oligomers formed *in vivo*, we assayed brain tissue homogenates from APP/PS1 rats expressing human amylin specifically in the pancreatic β-cells (*i.e.*, APP/PS1/HIP rats). APP/PS1 rats that express WT endogenous rat amylin were negative controls for the amyloidogenicity of human amylin. Full characterization of cerebral amylin-Aβ pathology and behavior deficits in APP/PS1/HIP *versus* APP/PS1 rats were recently published (12). Sandwich ELISA signal intensities were measured in APP/PS1/HIP and APP/PS1 brain tissue homogenates. The mean level of oligomerized amylin-Aβ is higher in APP/PS1/HIP brain tissue homogenates than those in the APP/PS1 rat group (Kruskal–Wallis one-way ANOVA, *p* < 0.01; Fig. 3*B*).

To assess the sensitivity of the ELISA to the fragmentation of amylin-Aβ hetero-oligomers, brain tissue homogenates from same rats were treated with 1 M hydrochloric acid (HCL) followed by the sandwich ELISA assay. Fragmenting of amylin-Aβ hetero-oligomers decreased sandwich ELISA signal intensities in APP/PS1/HIP rat brain tissue homogenates (Fig. 3*C*). Although the sandwich ELISA detects rat amylin-Aβ hetero-oligomers in rat amylin-Aβ solutions (Fig. 2*C*), the average detection intensities corresponding to rat amylin-Aβ hetero-oligomers in HCl-treated *versus* untreated APP/PS1 brain tissue homogenates are similar (Fig. 3*B*). This may suggest the presence of low concentrations of rat amylin-Aβ hetero-oligomers in APP/PS1 rat brains. In the same APP/PS1/HIP rat brain homogenates as in Figure 3*B*, fragmenting of amylin-Aβ hetero-oligomers appears associated with more solubilized amylin (Fig. 3*D*), although the analysis shows a large variability of the amylin ELISA signal intensity in in HCl-treated *versus* nontreated tissue homogenates (Fig. 3*E*).

Taken together, the results (Figs. 2 and 3) indicate that the amylin-Aβ sandwich ELISA measures the concentrations of co-aggregated amylin-Aβ (hetero-oligomers) in a linear fashion and is sensitive to the Th-T biochemical characteristics of early stage amylin-Aβ hetero-oligomers and the dissociation of amylin-Aβ hetero-oligomers to monomers.

### Amylin-Aβ hetero-oligomer levels in AD *versus* non-AD brains

By using the amylin-Aβ sandwich ELISA, we screened 76 human temporal cortex homogenates for amylin-Aβ hetero-oligomers. Our sample includes early onset fAD (n = 18), sAD (n = 45), and non-AD (n = 13) brains. The fAD group includes APP and PS1 mutation carriers and is the positive control for cerebral mixed amylin-Aβ pathology (12). The assessment of sAD-type dementia is based on the density of neuritic plaques (Consortium to Establish a Registry for Alzheimer’s Disease; CERAD) and Braak NFT stage, as previously described (12, 13, 26, 27, 28, 29, 30). The sAD group includes n = 42 brains that have CERAD A, B, or C and n = 3 brains that have CERAD *none* and higher Braak scores (III-IV). The non-AD group includes n = 12 brains that have CERAD none and low Braak scores (0, I, or II) and one brain that was scored with CERAD A and Braak NFT stage 0. Individuals in sAD and non-AD groups were of similar age at postmortem brain tissue collection (85.65 ± 7.04 years *versus* 87.22 ± 7.30 years; *p* = 0.482). Amylin and Aβ concentrations were measured in all brain tissue homogenates using conventional ELISAs.

**Figure 4:**
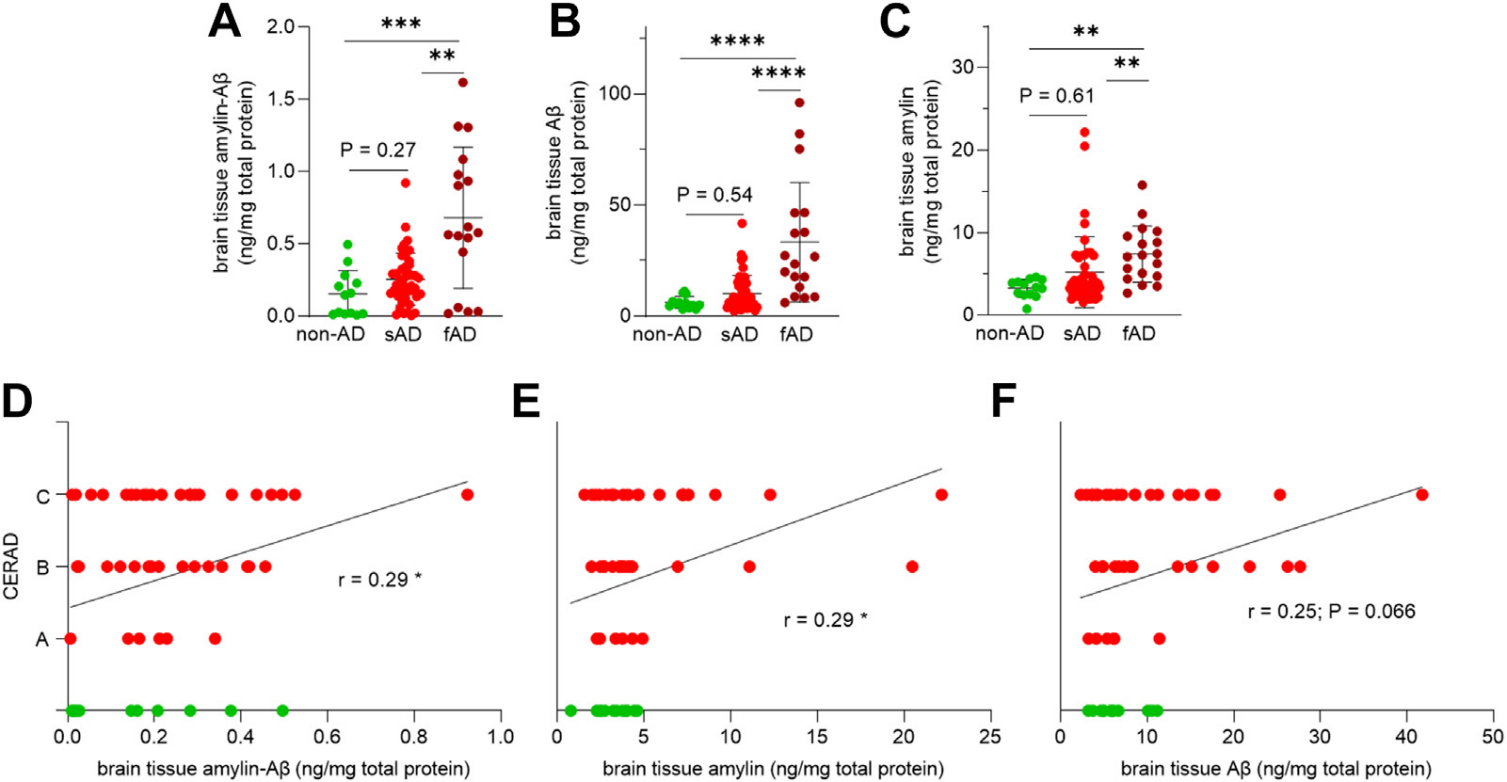
**Correlations between brain tissue amylin-Aβ, amylin, and Aβ concentrations with CERAD score of sAD brains.***A*–*C*, brain tissue amylin-Aβ concentrations (*A*) measured using the amylin-Aβ sandwich ELISA in APP and PS1 mutation carriers (fAD; n = 18), persons with sAD (n = 45), and non-AD individuals (n = 13). *D*–*F*, the pairwise correlation analysis of CERAD scores of sAD and non-AD brains *versus* amylin-Aβ concentrations (*D*), CERAD scores of sAD and non-AD brains *versus* brain tissue amylin concentrations (*E*), and CERAD scores of sAD and non-AD brains *versus* brain tissue Aβ concentrations (*F*). Analyzed brains have CERAD *none* (n = 12), CERAD A (n = 6), CERAD B (n = 15) and CERAD C (n = 22). n = 3 sAD brains that have divergent amyloid plaque scores (CERAD none and Braak stages III–IV) were not included in the pairwise correlation analysis. Data are mean ± SD. One-way ANOVA test in (*A*–*C*) ∗∗*p* < 0.01, ∗∗∗*p* < 0.001, and ∗∗∗∗*p* <0.0001. Data are presented as correlation analysis, Pearson’s correlation in (*D*–*F*), ∗*p* < 0.05. Aβ, β amyloid; AD, Alzheimer’s disease; APP, amyloid precursor protein; CERAD, Consortium to Establish a Registry for Alzheimer’s Disease; fAD, familial Alzheimer’s disease; PS1, presenilin 1; sAD, sporadic Alzheimer’s disease.

Frequency distributions of brain tissue amylin, Aβ, and amylin-Aβ hetero-oligomer concentrations indicate distinct homeostasis of amylin and Aβ amyloidogenic peptides within the fAD and sAD groups (Fig. S2). Most of the disease brains had detectable amylin-Aβ hetero-oligomer concentrations, whereas about half control subjects had brain tissue amylin-Aβ hetero-oligomers below our detection limit (0.02 ng/mg total protein) (n = 6 samples with no ELISA evidence of brain amylin-Aβ hetero-oligomers; Fig. S2*C*). The mean level of oligomerized amylin-Aβ is higher in brains within the fAD group than those in the sAD and non-AD groups (one-way ANOVA, *p* < 0.001 and *p* < 0.01, respectively Fig. 4*A*), consistent with similar relationships between the average amylin and Aβ levels in fAD compared to sAD and non-AD groups (Fig. 4, *B* and *C*). In the sAD group, the average Aβ and amylin levels are not significantly increased compared to those in the non-AD group (one-way ANOVA, *p* > 0.05, Fig. 4, *B* and *C*), which appears to explain why amylin-Aβ hetero-oligomer levels are only significantly increased in patients with fAD, but not sAD, relative to non-AD patients (Fig. 4*A*). Increased brain tissue amylin-Aβ hetero-oligomer, amylin, and Aβ levels are more common in brains that have CERAD A, B, or C, compared to those that have CERAD *none* (Fig. 4, *D*–*F*). The pairwise correlation coefficient suggests a possible relationship between brain tissue amylin-Aβ hetero-oligomer levels and frequent neuritic plaques (*r* = 0.29; *p* < 0.05) (Fig. 4*D*). n = 3 sAD brains that have scattered amyloid plaque scores (CERAD *none* and Braak stage III–IV) were excluded from the pairwise correlation analysis.

**Figure 5:**
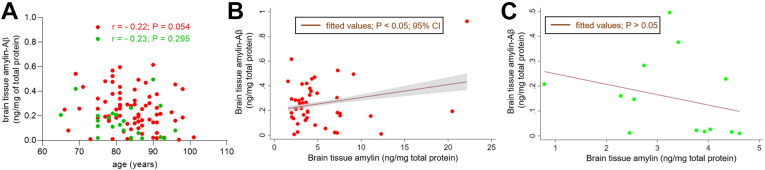
**The relationships between amylin-Aβ oligomerization and brain amylin and Aβ levels in age-related AD.***A*, pairwise correlation analyses of brain tissue amylin-Aβ concentrations *versus* age for sAD and non-AD groups. *B*, brain amylin-Aβ hetero-oligomer levels as a function of brain amylin levels. Regression line and confidence interval for the mean are shown for subjects in the AD group (n = 46). The *p* value shown was calculated by linear regression for the effect of brain amylin level on the amylin-Aβ hetero-oligomerization with adjustment for brain Aβ level (*p* = 0.05) and a multiplicative (amylin × Aβ) interaction term (*p* = 0.13, after including the main effect terms (brain amylin and Aβ levels)). *C*, same as in above for the non-AD group (n = 13). Aβ, β amyloid; AD, Alzheimer’s disease; sAD, sporadic Alzheimer’s disease.

Given the association between sAD pathology and advanced age (31), we assessed a potential relationship between cerebral amylin-Aβ hetero-oligomerization and age in sAD and non-AD groups. Overall, there is no correlation between cerebral amylin-Aβ hetero-oligomer accumulation and age (Fig. 5*A*). In sAD brains, however, there is an apparent propensity to lower levels of amylin-Aβ hetero-oligomer levels with increasing age (*p* = 0.054), which may reflect the demonstrated shift of Aβ to plaque formation during age-related AD pathogenesis (32).

### Relationships between amylin-Aβ hetero-oligomerization and brain amylin and Aβ levels in the setting of type 2 diabetes mellitus

**Figure 6:**
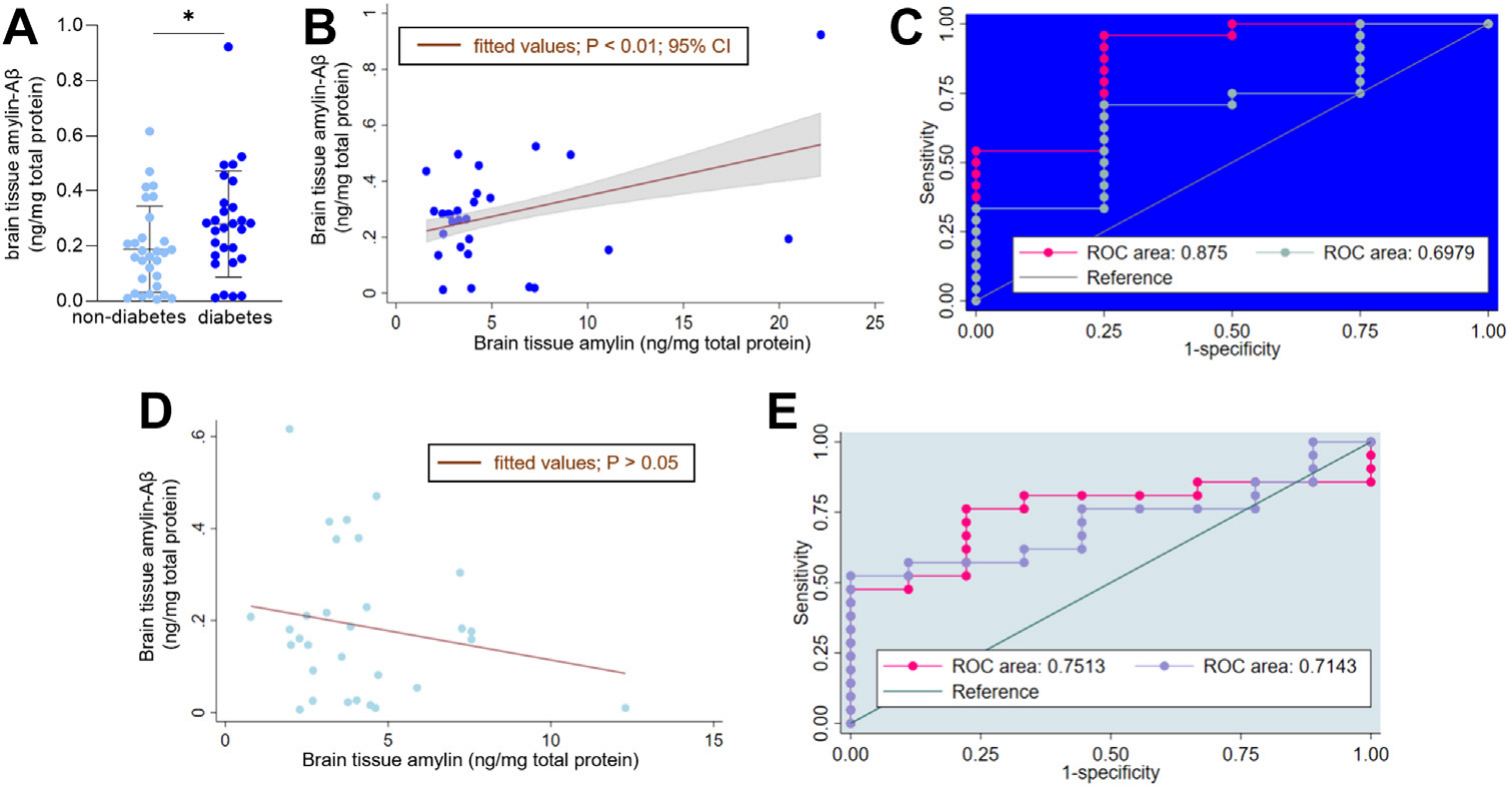
**The relationships between amylin-Aβ oligomerization and brain amylin and Aβ levels in the setting of type 2 diabetes.***A*, brain tissue amylin-Aβ concentrations measured using the amylin-Aβ sandwich ELISA in groups of individuals stratified by type 2 diabetes mellitus (diabetes group n = 28; nondiabetes group; n = 30). ∗*p* < 0.05. *B*, brain amylin-Aβ hetero-oligomer levels as a function of brain amylin levels. Regression line and confidence interval for the mean are shown for subjects in the diabetes group. The *p* value shown was calculated by linear regression for the effect of brain amylin level on the amylin-Aβ hetero-oligomerization with adjustment for brain Aβ level (*p* < 0.05) and a multiplicative (amylin × Aβ) interaction term (*p* < 0.05). *C*, goodness-of-fit test for a multiple logistic regression model based on brain amylin, amylin-Aβ, and age as covariates and a logistic model based on brain Aβ level and age as predictor variables. Receiver operating characteristic (ROC) curves were created plotting sensitivity *versus*—specificity for all possible cut points from fitted values in a model based on brain amylin, amylin-Aβ, and age (*pink curve*) *versus* a model based on brain Aβ level and age (*gray curve*). *D*, same as in (*B*), for the nondiabetes group. *E*, same as in (*C*), for the nondiabetes group. Aβ, β amyloid.

Dysregulated amylin is a contributing factor to the pathogenesis of both type 2 diabetes (6, 7, 8) and AD (9, 10, 11, 12, 13). We conducted regression analyses of the relationships between amylin-Aβ hetero-oligomerization and brain amylin and Aβ levels (predictor variables) in subjects stratified on the diagnosis of AD *versus* non-AD and diabetes *versus* nondiabetes (Figs. 5 and 6). Age and sex were included as covariates in all analyses (see Experimental procedures for detailed regression models).

Our previous data showing a direct relationship between amylin and Aβ levels in AD brains (12, 13) suggest a possible effect modification in which the effect of brain amylin level on the amylin-Aβ hetero-oligomerization may depend on the Aβ level. To test this hypothesis, we employed a multiple linear regression model that includes brain amylin and Aβ levels as predictor variables and a multiplicative (amylin × Aβ) interaction term. The interaction term is not associated with amylin-Aβ hetero-oligomerization (*p* = 0.13) after including the main effect terms (brain amylin and Aβ levels). Brain amylin-Aβ hetero-oligomerization is associated with brain amylin levels only in the AD group (Fig. 5, *B* and *C*). The regression coefficients are only marginally altered after adjusting for the brain Aβ levels (*p* = 0.05); however, we retained the Aβ term in the multiple linear regression analysis. In the AD group, the regression equation is Y = 0.1 + 0.03 amylin (*p* = 0.045) + 0.01 Aβ (*p* = 0.050). For a given brain Aβ level, each ng/mg increase of brain amylin level is associated with an average increase in amylin-Aβ hetero-oligomers of 0.03 ng/mg. The intercept is 0.1 and has no biological meaning as brain amylin and Aβ levels are nonzero.

In groups stratified based on the diabetes status, the average level of oligomerized amylin-Aβ is higher in those with type 2 diabetes mellitus (diabetes group; n = 28) *versus* those without (nondiabetes group; n = 30) (Fig. 6*A*). The linear regression model shows amylin-Aβ hetero-oligomerization is associated with increased brain amylin level (*p* = 0.006) after adjusting for the brain Aβ level (*p* = 0.023) and the interaction term (amylin x Aβ) is significant (*p* = 0.031), in the diabetes group. The regression equation is Y = 0.048 + 0.041 amylin + 0.012 Aβ − 0.002 amylin × Aβ (Fig. 6*B*). For given brain Aβ level and magnitude of amylin × Aβ interaction, each ng/mg increase of brain amylin level is associated with an average increase in amylin-Aβ hetero-oligomers of 0.041 ng/mg. The intercept has no biological meaning, as brain amylin and Aβ levels are nonzero. The interaction coefficient is negative, suggesting a reduction of effect modification of brain amylin level on the amylin-Aβ hetero-oligomerization at higher brain Aβ levels (*i.e.*, a possible saturation effect).

To assess the extent of amylin-Aβ hetero-oligomerization effect on the link between AD and type 2 diabetes, we used logistic regression models based on brain amylin, Aβ, and amylin-Aβ hetero-oligomer levels as covariates. We then compared the goodness-of-fit of the model in persons with AD and diabetes *versus* persons with AD without diabetes. A model based on brain amylin, amylin-Aβ, and age as covariates has a >15% increase of the area under the receiver operating characteristic (ROC) curve (pink curve, ROC area = 0.875; Fig. 6*C*) compared to the ROC of a logistic model based on brain Aβ level and age as predictor variables (gray curve, ROC area = 0.698; Fig. 6*C*). Consistent with this result, our data show improved confidence interval (1.0–1.2) for the odds ratio (1.1; *p* = 0.031) corresponding to the amylin-Aβ-based model, compared to the confidence interval (0.98–1.3) for the odds ratio = 1.14 (*p* = 0.088) corresponding to the Aβ-based model. Therefore, the amylin-Aβ–based model improves the prediction of AD/non-AD status, compared to a logistic model based on the Aβ-based model, in the setting of type 2 diabetes.

In contrast to the diabetes group, in which the regression coefficients of the linear regression model are significant (Fig. 6*B*), the relationship between brain amylin-Aβ hetero-oligomerization and brain amylin levels adjusted for the brain Aβ levels is not significant (*p* < 0.05) (Fig. 6*D*), in persons without diabetes. Furthermore, ROC areas corresponding to the two logistic regression models (*i.e.*, amylin-Aβ *versus* Aβ-based models) are similar (*i.e.*, 0.751 *versus* 0.714; Fig. 6*E*), in the nondiabetes AD group. Either brain amylin-Aβ or Aβ-based models similarly predict AD/non-AD status in persons without diabetes.

### Circulating amylin-Aβ hetero-oligomers in blood

**Figure 7:**
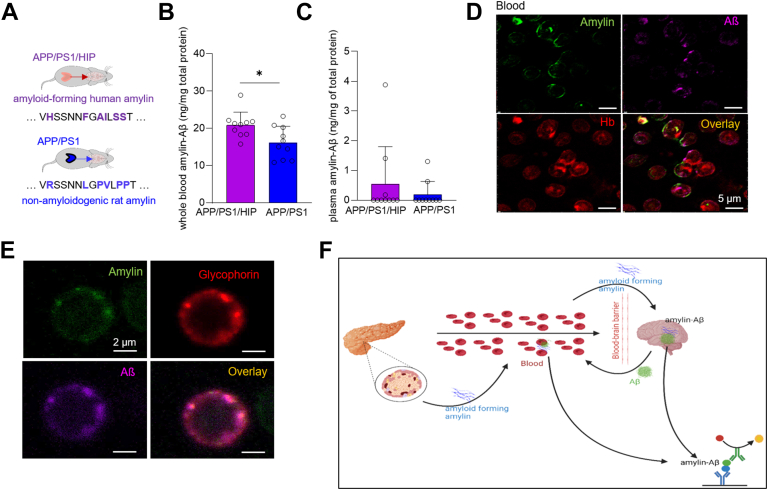
**Detection of amylin-Aβ hetero-oligomers in the blood sampled from APP/PS1/HIP and APP/PS1 rats.***A*, the illustration shows differences in the amino acid sequences of human *versus* rat amylin peptides. *B* and *C*, amylin-Aβ hetero-oligomer concentrations measured in whole blood (*B*) and plasma (*C*) from age-matched APP/PS1/HIP and APP/PS1 rats (age, 16 months, n = 10 males/group) by using the amylin-Aβ sandwich ELISA. *D* and *E*, representative images of confocal microscopic analysis of red blood cells from APP/PS1/HIP rats (n = 5) triple-stained with anti-amylin antibody (*green*), anti-Aβ antibody (*magenta*), and hemoglobin (*red*) (*D*), as well as triple-stained with anti-amylin antibody (*green*), anti-Aβ antibody (*magenta*), and glycophorin (*red*) (*E*). *F*, schematic summary of the results: amyloid-forming human amylin secreted from the pancreas into the blood forms mixed amylin-Aβ hetero-oligomers that accumulates in circulating RBCs and within the brain that can be detected by the amylin-Aβ sandwich ELISA. Data are means ± SD, unpaired two-tailed *t* test ∗*p* < 0.05 in (*B*); Scale bars represent 2 μm in (*D*) and 2 μm in (*E*). Aβ, β amyloid; APP, human islet amyloid polypeptide; HIP, human islet amyloid polypeptide; PS1, presenilin 1; RBC, red blood cell.

Using the amylin-Aβ sandwich ELISA, we measured amylin-Aβ oligomer levels in whole blood lysates from APP/PS1 rats expressing amyloid-forming amylin in the pancreas (male APP/PS1/HIP rats; age, 16 months). Whole blood lysates from male APP/PS1 littermates were the negative controls for amyloidogenicity of human amylin. Our results show increased levels of amylin-Aβ hetero-oligomers in whole blood lysates obtained from APP/PS1/HIP rats compared to those from APP/PS1 littermates (Fig. 7*A*). Plasma amylin-Aβ ELISA indicates amylin-Aβ hetero-oligomers were below the detection limit of our ELISA in most samples (Fig. 7*B*). Figure 7*C* shows representative confocal microscopic images of red blood cells (RBCs) from APP/PS1/HIP rats; samples were triple-stained for amylin, Aβ, and hemoglobin, a protein that is specific for RBCs. To further assess the potential amylin-Aβ colocalization on the RBC membrane, RBC samples were triple-stained for amylin, Aβ, and glycophorin (Fig. 7*D*), a protein that is expressed within the RBC membrane. Taken together, the results suggest amylin-Aβ codeposits on circulating RBCs. Circulating amylin-Aβ hetero-oligomers and those accumulating within brain tissues have shared prefibrillar molecular structures and can be detected by the amylin-Aβ ELISA (Fig. 7*E*).

## Discussion

Accumulating data from studies in humans suggest an association between pancreatic amyloid-forming amylin and cerebral Aβ pathology (9, 10, 11, 12, 13). Our results indicate cerebral amylin-Aβ plaques form through mechanisms that involve amylin-Aβ co-aggregation at the molecular level. We show that amylin-Aβ hetero-oligomers can be measured by an amylin-Aβ sandwich ELISA. The utility of this assay is demonstrated by the analysis of postmortem brain tissues collected from cognitively unaffected individuals and persons with AD. Using transgenic AD-model rats, we show that this new assay can detect circulating amylin-Aβ hetero-oligomers in the blood and is sensitive to the dissociation of amylin-Aβ hetero-oligomers to monomers.

The sensitivity of the sandwich ELISA to detect amylin-Aβ hetero-oligomers depends on matching of the capture and detection antibody pairs to recognize exposed epitopes that are distinct from amylin-Aβ binding sites on both amylin and Aβ peptides. The antibody against total Aβ recognizes an epitope distinct from the high affinity amylin-Aβ binding sites, which facilitates the increased sensitivity of the sandwich amylin-Aβ ELISA assay when carried out using anti-total Aβ antibody as the detection antibody. In contrast, the antibodies against the Aβ isoform ending at the 42nd amino acid (Aβ_42_ antibody) and the isoform ending at 40th amino acid (Aβ_40_ antibody) recognize epitopes that appear involved in amylin-Aβ co-aggregation. The Aβ_42_ and Aβ_40_ antibodies appear less sensitive to amylin-Aβ hetero-oligomer detection. Based on our results and previous analyses of amylin-Aβ oligomerization *ex vivo* (14, 16), we concluded that the N-terminal epitope of amylin may not be involved in the amylin-Aβ aggregate formation. Related to this point, human amylin differs from rodent amylin by six amino acids at the C-terminus (25). Because of the similarity between human and rat amylin, the polyclonal P2 N-terminus amylin antibody cross-reacts with rat amylin, which may explain the presence of a weak Th-T fluorescence signal intensity detected in the rat amylin-Aβ aggregates. Consistent with this result and previously published data (20), amylin-Aβ ELISA signal intensities in human amylin-Aβ co-aggregation assays are about 7-fold higher than that corresponding to rat amylin-Aβ co-aggregation.

The evolution of the sandwich ELISA signal in an incubated solution of amylin and Aβ peptides analyzed in parallel for amyloid fibril formation *via* measurements of Th-T fluorescence indicates that the sandwich ELISA may detect prefibrillar amylin-Aβ hetero-oligomers. In APP/PS1/HIP rats, circulating amylin-Aβ hetero-oligomers and those accumulating within brain tissues appear to share prefibrillar molecular structures that can be detected by the amylin-Aβ ELISA. Both human amylin and Aβ have high propensity to attach to cellular membranes (33, 34, 35), which is shown by amylin-Aβ codeposition on circulating RBCs. Amylin deposition on RBCs is common in persons with diabetes-related microvascular complications (36). We hypothesize that amylin-Aβ codeposits on circulating RBCs in APP/PS1/HIP rats reflect amylin-Aβ hetero-oligomerization within the microvasculature. In the rodent model of APP/PS1 dementia (37), the reported level of Aβ in the blood is in the ∼10 to 70 ng/ml range. Given that APP/PS1/HIP rats have about 2-fold increase of blood amylin compared to APP/PS1 littermates (12), we remain cautious in interpreting differences in amylin-Aβ levels determined for APP/PS1/HIP *versus* APP/PS1 rats solely on the basis of amyloidogenicity of human amylin. Studies are needed to determine the extent to which amylin-Aβ hetero-oligomerization can be detected in human blood.

Previous studies showed that amylin concentrations in blood and cerebrospinal fluid are higher in AD than in cognitively unaffected persons (12, 13, 38, 39), suggesting a possible relationship between circulating amylin levels and the propensity of amylin to accumulate in the brain. Because of the accumulating evidence suggesting that pancreatic amyloid-forming amylin synergistically co-aggregates with vascular and parenchymal Aβ (9, 10, 11, 12), it is important to be able to specifically measure molecular amylin-Aβ species in a range of biological samples. A lack of rapid, accessible, scalable, and accurate biochemical methods to quantify amylin-Aβ interaction prevents larger sample studies, hindering the development of therapeutic strategies to prevent brain amylin accumulation and interaction with AD pathology. Our results confirm that amylin-Aβ hetero-oligomers can be detected and quantified and may serve as a marker of amylin-Aβ established interaction and its correlation with AD pathology.

In groups stratified based on the diabetes status, the average level of amylin-Aβ hetero-oligomerization is higher in those with type 2 diabetes mellitus. The slopes with respect to brain amylin levels appear different between the groups, suggesting that, in a larger cohort that includes individuals without diabetes, there may occur effect modification, and the measures of association in the subgroups may differ from one another. We found a significant relationship between brain amylin-Aβ hetero-oligomerization and brain amylin levels adjusted for the brain Aβ levels, in the diabetes group. Potential mechanisms accounting for diabetes-associated increased amylin-Aβ hetero-oligomerization may involve hypersecretion of amyloid-forming pancreatic amylin (2). The interaction coefficient is negative, suggesting a reduction of effect modification of brain amylin level on the amylin-Aβ hetero-oligomerization at higher brain Aβ levels (*i.e.*, saturation effect). In the diabetes group, the logistic regression model based on brain amylin and amylin-Aβ hetero-oligomer levels as covariates improves the prediction of AD/non-AD status, compared to a logistic model based on brain Aβ level and age as predictor variables. Taken together, our results suggest that it may be important to account for brain tissue amylin-Aβ hetero-oligomerization in assessing the effect of diabetes on AD.

Limitations of current amylin-Aβ ELISA include a lack of structural and component information on the detected hetero-oligomer (specific numbers of amylin and Aβ monomers making up the hetero-oligomer). Another limitation is the need of sample thawing at room temperature. The duration of sample thawing may affect the concentration of amylin-Aβ hetero-oligomers because the process of aggregation of amyloid-forming peptides or fibrils is continuous, and some hetero-oligomers could disintegrate over time.

In conclusion, future research should focus on the detection of circulating amylin-Aβ hetero-oligomers in a clinical setting of AD. This is important because assessing dynamics of amylin-Aβ interaction during AD pathogenesis may provide clues for potential therapeutic interventions when amylin-Aβ molecular processes can be modulated effectively.

## Experimental procedures

### Anti-amylin antibody generation

Immunization of New Zealand White rabbit was performed with purified N-terminal synthetic amylin peptide (5′-CKCNTATCATQRLANFLVHSS-3′) conjugated with keyhole limpet hemocyanin antigen as described by Saradhi *et al.* (40) with some modifications. Briefly, the rabbit was immunized subcutaneously at four different sites with 1 mg of immunogen after suspending in equal volume of complete Freund’s adjuvant (0.5 ml). Every 3 weeks after initial immunization, the rabbit was injected with 1 mg of amylin with alum adjuvant as booster injections for a total of three booster doses administered. Preimmunization blood was collected before the first dose of injection and before every booster dose from the rabbit’s central artery of ear under sedation. Three weeks after the final booster dose, blood was collected by cardiac puncture under full anesthesia. The blood serum was isolated and further purified by Protein A Plus Spin Columns (NAb, 89956, Thermo Fisher Scientific) followed by characterization of P2 amylin antibody with IP, Western blot, ELISA, and immunohistochemistry. The characterization of the anti-amylin P2 antibody is detailed in Fig. S4.

### Amylin-Aβ sandwich ELISA

Amylin-Aβ sandwich ELISA was used to measure the concentrations of amylin-Aβ in human/rat brain homogenates. Briefly, a 96-well plate was coated with 100 μl of mouse anti-human-Aβ (1:400; clone 6E10, 803002, Biolegend) antibody for standard and with rabbit anti-amylin P2 antibody (1:400; 2 mg/ml stock) for samples in bicarbonate buffer (0.028 M Na_2_CO_3_, 0.071 M NaHCO_3_, pH 9.6) as coating antibodies overnight at 4 °C temperature. The next day, plate was washed 1 to 2 times with 300 μl of washing buffer [PBS with 0.05% of Tween 20 (0.05% PBST)] and blocked by 300 μl of assay diluent (421203, Biolegend) for 1 h at room temperature. Again, the plate was washed with 0.05% PBST washing buffer and incubated with 100 μl of Aβ_40_/Aβ_42_ standards and brain homogenate samples overnight at 4 °C. Assay diluent/PBS was used as a blank for standard and sample wells. The next day after three washing cycles, the plate was incubated with 100 μl of either mouse anti-human-total-Aβ (1:400, clone-4G8, 800720—Biolegend), mouse anti-human-Aβ_42_ (1:400, 805507, Biolegend; specific for the isoform ending at the 42nd amino acid), or mouse anti-human-Aβ40 (1:400, 805407, Biolegend; specific for the isoform ending at the 40th amino acid) conjugated-HRP detection antibody for 1 h at room temperature. After completing the incubation, the plate was washed 3 to 4 times followed by incubation with 100 μl of 3,3′,5,5′ tetramethylbenzidine substrate (34028, Thermo Fisher Scientific). The reaction was stopped by adding 50 μl of stop solution (N600, Thermo Fisher Scientific) after developing the signals; the plate was read at 450 nm in a spectrophotometer. Amylin-Aβ concentrations were calculated by generating a graph between the relative absorbance (A_450_) of standards *versus* standard concentrations, after subtracting the absorbance of standard blank and sample blank from the absorbance of standards and samples, respectively.

The LOD of the amylin-Aβ sandwich ELISA (0.02 ng/mg of total protein) is calculated as reported by Armbruster *et al.* (41). LOD is the lowest analyte concentration likely to be reliably distinguished from the limit of blank (LOB) at which detection is feasible. LOD is determined by utilizing both the measured LOB and test replicates of a sample known to contain a low concentration of analyte: LOD = LOB + 1.645∗(SD low concentration sample). LOB is the highest apparent analyte concentration expected to be found when replicates of a blank sample containing no analyte are tested: LOB = mean blank + 1.645∗(SD blank), where SD is the standard deviation.

In a separate experiment, commercially available amylin antibodies (1:400, T-4157, Bachem-Peninsula Laboratories; 1:400, SC-377530, E5 Santa Cruz; 1:400, abx-324813, Abbexa; 1:400, 70R-15263, Fitzgerald) were used as coating antibody to compare with the rabbit anti-amylin P2 antibody in amylin-Aβ sandwich ELISA.

### Conventional amylin and Aβ ELISAs

Indirect ELISA was used to measure the concentrations of amylin in human brain, rat brain, and pancreas homogenates as described by Kohl *et al.* (42) with some modifications. Briefly, a 96-well plate was coated with 50 μl of bicarbonate buffer with 50 μl of amylin standard and brain homogenate samples overnight at 4 °C temperature. The next day, the plate was washed with 300 μl of washing buffer [tris buffer saline with 0.05% of Tween 20 (0.05% TBST)] and blocked by 300 μl of assay diluent for 1 h at room temperature. Again, the plate was washed with 0.05% TBST washing buffer and incubated with 100 μl volume of rabbit anti-amylin P2 detection antibody (1:400) overnight at 4 °C temperature. The next day, the plate was washed three times with washing buffer and incubated with anti-rabbit IgG HRP-conjugated (1:400; NA934VS; GE Healthcare) antibody for 1 h at room temperature. The plate was washed 3 to 4 times followed by incubation with 100 μl of 3,3′,5,5′ tetramethylbenzidine substrate. The reaction was stopped by adding 50 μl of stop solution after getting the signals, and the plate was read at 450 nm in a spectrophotometer. For comparison, a commercially available rabbit anti-amylin antibody (1:400, T-4157, Bachem-Peninsula Laboratories) was also used as a detection antibody in rat pancreas homogenate.

To measure the titer of rabbit anti-amylin P2 antibody, an indirect ELISA was used. The end point dilution method was used to determine the titer of amylin P2 antibody. Antibody titers were defined as the reciprocal of the highest amylin P2 antibody dilution that produced an absorbance value above the blank.

Amylin ELISA (EZHA-52K, Millipore Sigma) was used to measure the amylin concentrations in brain homogenates. Aβ_42_ concentrations in brain homogenates were measured using the sandwich ELISA Kit for Aβ_42_ (Thermo Fisher Scientific, cat # KHB3441). Concentrations of target proteins were normalized to the amount of total protein input assessed using the bicinchoninic acid method in all experiments.

### Human samples

This research employed de-identified frozen brain tissue from the biobank of the Alzheimer’s Disease Research Center at the University of Kentucky (UK-ADRC) under a protocol approved by the University of Kentucky Institutional Review Board. Informed consent was obtained prospectively. Frozen temporal cortex tissue samples were obtained from 45 persons with sAD-type dementia documented by Aβ positivity and 13 cognitively unaffected individuals. Patient characteristics including cognitive status, sex, diabetes status, and age, along with clinical and neuropathological information are described in Table 1. The absence/presence of diabetes was determined during life (at longitudinal clinical visits) by patient or caregiver self-report and the use of diabetic medications. The assessment of clinical dementia and the neuropathologic features—neuritic amyloid plaques (CERAD) and Braak NFT stage—were scored as previously described (26, 27, 28, 29, 30). Amylin-Aβ concentrations were measured in fAD brains (n = 18) with previously documented amylin accumulation through immunohistochemistry and ELISA (12). Frozen temporal cortex tissues from fAD mutation carriers were provided by the Queen Square Brain Bank for Neurological Disorders at UCL Queen Square Institute of Neurology (United Kingdom) and King’s College London (United Kingdom). Patient characteristics were previously described (12).

**Table 1:** Summary statistics for individuals within sAD and non-AD groups

	AD	Non-AD
Brain samples (ELISA)	*n = 45*	*n = 13*
Gender, female/male (% female)	23/22 (51.1)	10/3 (76.92)
Age at collection in years (avg ± SEM)	85.65 ± 7.04	87.22 ± 7.30
Type 2 diabetes (%)	51.06	38.46

Abbreviations: AD, Alzheimer’s disease; sAD, sporadic Alzheimer’s disease.

### Experimental animals

This investigation conforms to the Guide for the Care and Use of Laboratory Animals published by the US National Academies Press (eighth edition, 2011) and was approved by the Institutional Animal Care and Use Committee at the University of Kentucky. We used transgenic rats (n = 26) and rabbits (all males). New Zealand white rabbits aged 2 to 3 months (Charles River Labs; n = 2) were used for immunization to generate rabbit anti-amylin P2 polyclonal antibody. Rats were housed in individually ventilated cages, on a 12-h light cycle and received a standard pelleted diet and water *ad libitum*. APP/PS1/HIP rats (n = 10 males; age 16 months) with APP/PS1 rats (n = 10 males; age 16 months) were used in measurements of amylin-Aβ concentrations in brain tissues and blood by using the amylin-Aβ sandwich ELISA. APP/PS1/HIP rats were generated as previously described (12). Briefly, TgF344-19 rats from the Rat Resource and Research Center, University of Missouri (APP/PS1 rats) are Fischer rats that express human Aβ (A4) precursor protein (hAPP) gene with the Swedish mutation (K595N/M596L) and presenilin 1 (PSEN1) gene with a deletion of exon 9, driven by mouse prion promoter (Prp). The APP/PS1 rats were crossbred with HIP rats to generate rats that are triple transgenic for human amylin, APP, and PSEN1 (APP/PS1/HIP rats). APP/PS1 rats that express nonamyloidogenic rat amylin served as controls for the amyloidogenic human amylin effects. Amylin knockout (AKO) rats that were characterized previously (43) were used as negative controls for amylin in biochemical studies.

### Tissue homogenization

Frozen human brain, rat brain, and pancreas tissues were homogenized in homogenate buffer (150 mM NaCl, 50 mM Tris–HCl, 50 mM NaF, 2% Triton X-100, 0.1% SDS, 1% (v/v) protease and phosphatase inhibitors, pH 7.5). The homogenates were left on ice for 15 min. Homogenates were centrifuged at 12,000*g* for 20 min at 4 °C. The supernatant was separated from pellet after centrifugation and then used for all experiments. For monomeric amylin, 100 μl of rat brain homogenates supernatants were treated with 20 to 30 μl of 1 M HCl acid for 30 min at room temperature and then incubated on ice for 15 min. By adding 5 to 10 μl 1 M Tris pH was neutralized and used in amylin-Aβ sandwich ELISA.

### Amylin-Aβ aggregate formation and the Thio-T assay for amyloid

Amylin-Aβ aggregates were prepared using the procedure described by O'Nuallain *et al.* (14). Briefly, 30 μM human amylin synthetic peptide (AS-60254-1, Anaspec) was incubated in PBSA (PBS + 0.05% sodium azide) in a water bath at 37 °C for 5 h. Aβ40 synthetic peptide (16.2 μM) (AS-24235, Anaspec) was preincubated at 37 °C for 2 h. The two solutions (1:1 volume) were then incubated in a water bath at 37 °C for 24 h. Aggregated amylin-Aβ was used in amylin-Aβ sandwich ELISA.

For Thio-T assay, 10 μl of solutions of prepared peptide aggregates were added in 96-well plate with 100 μl of 0.08 mg/ml Thio-T (Sigma). The plate was incubated at 37 °C for 1 min and fluorescence at 440 nm excitation and 482 nm emission was measured.

### Immunohistochemistry and immunofluorescence

For immunohistochemistry, formalin-fixed, paraffin-embedded brain tissues from HIP and AKO rats were processed as described before (12, 43). Rabbit anti-amylin P2 antibody was the primary antibody (dilution 1:100). Biotinylated anti-rabbit IgG (1:300, BA-1100, Vector) was the secondary antibody. Pancreas tissues from AKO rats were the negative control for amylin.

In immunofluorescence experiments, we used formalin-fixed, paraffin-embedded human brain tissue processed as previously described (12, 36, 43, 44). Anti-amylin (1:200; clone E5; SC-377530; Santa Cruz, and 1:200; T-4157, Bachem-Peninsula Laboratories) and anti-Aβ (1:400; clone 6E10, 803002, Biolegend) were the primary antibodies. Secondary antibodies were Alexa Fluor 488 conjugated anti-mouse IgG (1:300; A11029; Invitrogen), Alexa Fluor 568 conjugated anti-rabbit IgG (1:200; A11036; Invitrogen), Alexa Fluor 647 conjugated anti-mouse (#A21236, Invitrogen), and Alexa Fluor 568 conjugated anti-mouse IgG (1:300; A11004; Invitrogen). For Thio-S staining, after secondary antibody incubation, brain slides were incubated in 0.5% Thio-S for 30 min at room temperature. Slides were then incubated for 3 min in 70% ethanol, 5 min in 0.2% Sudan black before washing and mounting. Immunofluorescence was performed as described previously (12, 36, 43, 44).

### Proximity ligation assay

Formalin-fixed and paraffin-embedded sections (10 μm) from brain were rehydrated and pretreated with 95% formic acid for 3 min at ambient temperature to expose antigens. After rinsing in 50 mmol/L Tris–HCl buffer with 150 mmol/L NaCl (pH 7.4), brain sections were incubated with primary antibodies rabbit anti-human-amylin (1:200, anti-human-amylin; T-4157; Peninsula Laboratories) and mouse anti-Aβ antibody 6E10 (1:200, 803002, Biolegend) overnight at 4 °C. Duolink *in situ* PLA (Duolink *in situ* PLA, DUO92004, Sigma) was performed according to the manufacturer's protocol and previously described in detail (10, 44). Briefly, for detection of primary antibody pairs, sections were incubated for 90 min with oligonucleotide-conjugated anti-mouse IgG MINUS and anti-rabbit IgG PLUS (PLA probes) diluted 1:6 in Tris-buffered saline at 37 °C. Amplified DNA strands were detected with oligonucleotides conjugated to a fluorophore and nuclei stained with Hoechst dye.

### Immunoprecipitation

We used a previously published protocol (43). Briefly, rat pancreas homogenates (1000 μg of total protein) and amylin-Aβ (30 μM amylin and 16.2 μM Aβ; 1:1) aggregates were incubated with rabbit anti-amylin P2 antibody (1:25, 2 mg/ml stock) and normal rabbit Ig G (#2729; Cell Signaling Technology) as a control, overnight with end-over-end rotation, at 4 °C. Antigen–antibody complex was added to Immobilized Protein A/G resin slurry (20422, Thermo Fisher Scientific) for 2 h at ambient temperature, washed with wash buffer (5 mM of EGTA, 50 mM of Tris, 1% v/v of Triton X-100, pH 7.5 + 1% v/v protease inhibitor, 1% v/v phosphatase inhibitor) and samples eluted with elution buffer (1.5% w/v of glycine, 8% v/v of 1 N HCl, pH 2–3) from the resins using elution buffer. The eluate was used for Western blot analysis.

### Western blot

Western blot analysis was performed on amylin-Aβ IP eluates, rat pancreas IP eluates, and pancreas tissue homogenate from rats. Tissues were processed as described previously (1, 2, 3, 6, 9). Briefly, rat brain/pancreas tissue homogenates were prepared in homogenate buffer. The homogenates were left on ice for 15 min and centrifuged at 12,000*g* for 20-min. The supernatant was separated from the pellet after centrifugation and then used for Western blotting. Total protein levels were estimated using a BCA kit (23225, Thermo Fisher Scientific). Rabbit anti-amylin P2 (1:400), Rabbit anti-amylin polyclonal (1:2000; T-4157, Bachem-Peninsula Laboratories), mouse anti human-Aβ (1:400; clone 6E10, 803002, Biolegend) antibody were primary antibodies. Rat brain homogenates or immunoprecipitated rat amylin from pancreas homogenates or amylin-Aβ aggregates and IP eluates (50 μg of protein from tissue homogenate or immunoprecipitated amylin elution) were loaded on 8% SDS-PAGE gels. Amylin was resolved in SDS-PAGE. anti-rabbit IgG HRP conjugated (1:30,000; NA934VS; GE Healthcare) and anti-mouse IgG HRP-conjugated (1:20,000; NXA931; GE Healthcare) were secondary antibodies.

### Statistical analysis

The number of samples or animals in each analysis, the statistical analysis performed, and *p* values are reported in figures and figure legends. D'Agostino-Pearson and Kolmogorov–Smirnov test was used to test the normality distribution of continuous variables. Parametric comparisons of continuous variables with normal distributions were performed using two-tailed unpaired *t* test. Welch’s correction was used with *t* test to account for unequal variance from unequal sample sizes, if necessary. Parametric comparisons of three groups or more group means were performed using one-way or two-way ANOVA with the Bonferroni posttest. Relationships between two continuous variables were analyzed by correlation analysis. Data are presented as mean ± SD or as box and whisker plots. Difference between groups was considered significant when *p* < 0.05. Part of analyses were performed using GraphPad Prism 8.1 software (https://www.graphpad.com/).

### Regression models

STATA BE 17 was used for conducting multiple linear and logistic regression models. We employed regression analyses of the relationships between amylin-Aβ hetero-oligomerization and brain amylin and Aβ levels (predictor variables) in persons stratified on diagnosis of AD *versus* non-AD and diabetes *versus* nondiabetes. Age and sex were included as covariates in all analyses. To assess possible multicollinearity, the variance inflation factor (VIF) post estimation test was conducted for each multiple linear regression model. Models with VIF >10 were discarded. In our multiple linear regression model (Fig. 6*B*), the mean VIF is 4.19, indicating moderate correlation between the amylin and Aβ predictor variables. Residual plots were calculated for all models to detect outliers, assess homoscedasticity, and need for transformations. In the linear regression analyses, including age as a covariate leads to no significant change of the regression coefficients (*p* > 0.05); same for the sex covariate (*p* > 0.05). Residual plots did not detect significant outliers (Fig. S3, *A* and *B*); however, transformations of amylin and Aβ covariates (Fig. S3, *C* and *D*) may improve the two linear regression models shown in Figures 5*B* and 6*B*.

The logistic regression models included age and brain amylin, Aβ, and amylin-Aβ levels as covariates and were used for generating ROC curves and calculating the areas under the curves. The Hosmer–Lemeshow goodness of fit was used to test the fit in each model. Akaike's information criterion (AIC) and Bayesian information criterion (BIC) were used to compare the models. For example, in Figure 6*C*, STATA calculated AIC = 23.18 and BIC = 28.50 for the logistic regression model corresponding to amylin-Aβ–based model and AIC = 26.75 and BIC = 30.75 for the logistic regression model corresponding Aβ-based model, indicating the superiority of the former.

## Data availability

All of the data are contained within the article and the supporting information.

## Supporting information

This article contains supporting information (12).

## Conflict of interest

The authors declare that they have no conflicts of interest with the contents of this article.
