# Supplementary material for: Rapid, scalable assay of amylin-β amyloid co-aggregation in brain tissue and blood
Source: J Biol Chem. 2023 Apr 6;299(5):104682. doi: 10.1016/j.jbc.2023.104682 (PMC10192925; doi:10.1016/j.jbc.2023.104682)
Supplement: Supporting Figures S1–S4 [file mmc1.docx]

**Rapid, scalable assay of amylin-β amyloid co-aggregation in brain tissue and blood**

**Authors:** Deepak Kotiya^1,2^, Noah Leibold^1,2^, Nirmal Verma^1,2^, Gregory A. Jicha^3,4^, Larry B. Goldstein^4^, and Florin Despa^1,2,4^*


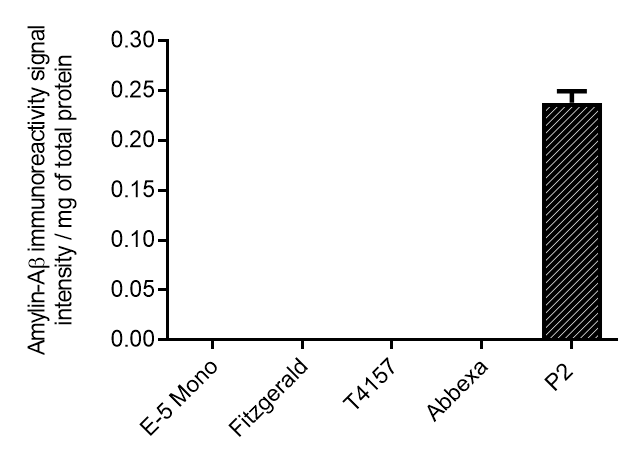


**Supplemental Figure S1.** Comparative analyses of amylin-Aβ immuno-reactivity signal intensities measured in amylin-Aβ solution phase (30μM amylin and 16.2 μM Aβ; 1:1) using amylin-Aβ sandwich ELISAs with the total Aβ antibody as the detection antibody and commercially available amylin antibodies (E-5, Fitzgerald, T4157, Abbexa) or P2 anti-amylin antibody as the capture antibody.

**
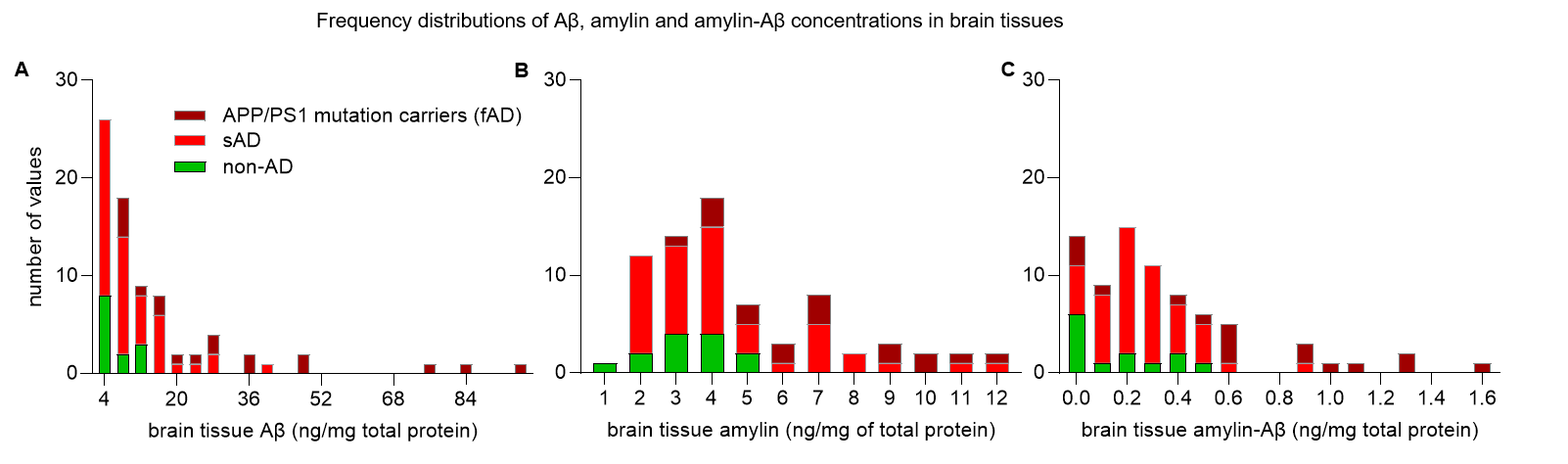
**

**Supplemental Figure S2. Frequency distributions of amylin, Aβ, and amylin-Aβ hetero-oligomers concentrations in human brain tissues.** *A-C,* Frequency distributions as the number of values of Aβ *(A)*, amylin *(B)***,** and amylin-Aβ hetero-oligomers *(C)* concentrations in the brains of *APP* and *PS1* mutation carriers (*n=18*), sporadic AD (sAD, *n=46*) and cognitively unaffected (CU; *n=13*) individuals. Data are means ± S.D. Data are presented as histograms in *(A-C)* or box and whiskers analyses in *(D-E)*.

**
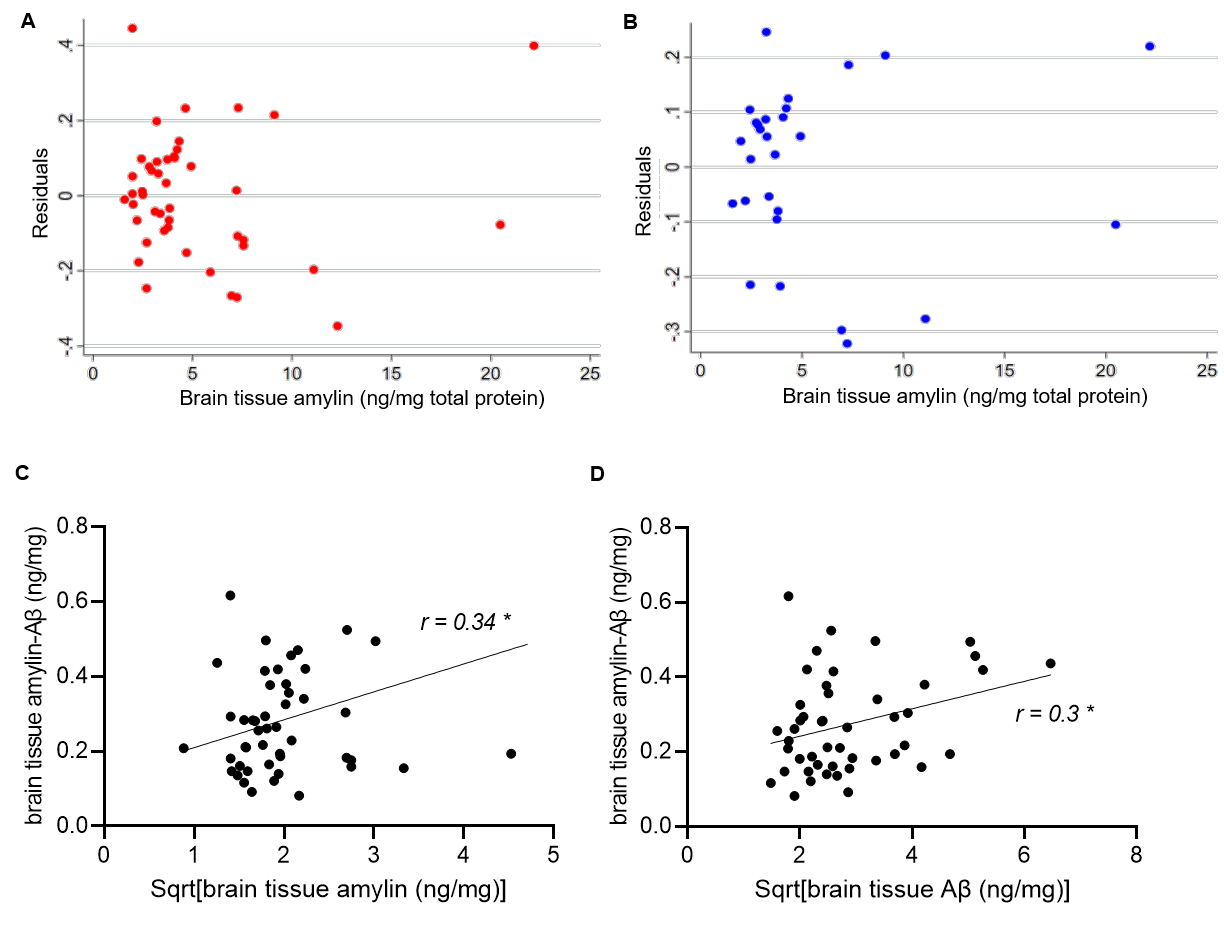
**

**Supplemental Figure S3**. A, Residual plot analysis calculated by linear regression for the effect of brain amylin level on the amylin-Aβ hetero-oligomerization with adjustment for brain Aβ level and a multiplicative (amylin x Aβ) interaction term (see Fig. 5B) in the AD group. B, Same as in above for the diabetes group (see Fig. 6B). *C-D,* Pairwise correlation analyses of brain tissue amylin-Aβ vs. brain amylin concentrations *(C)* and brain tissue amylin-Aβ vs. Aβ concentrations *(E)* within the sAD group. Data are presented as correlation analysis, Pearson’s correlation**.**


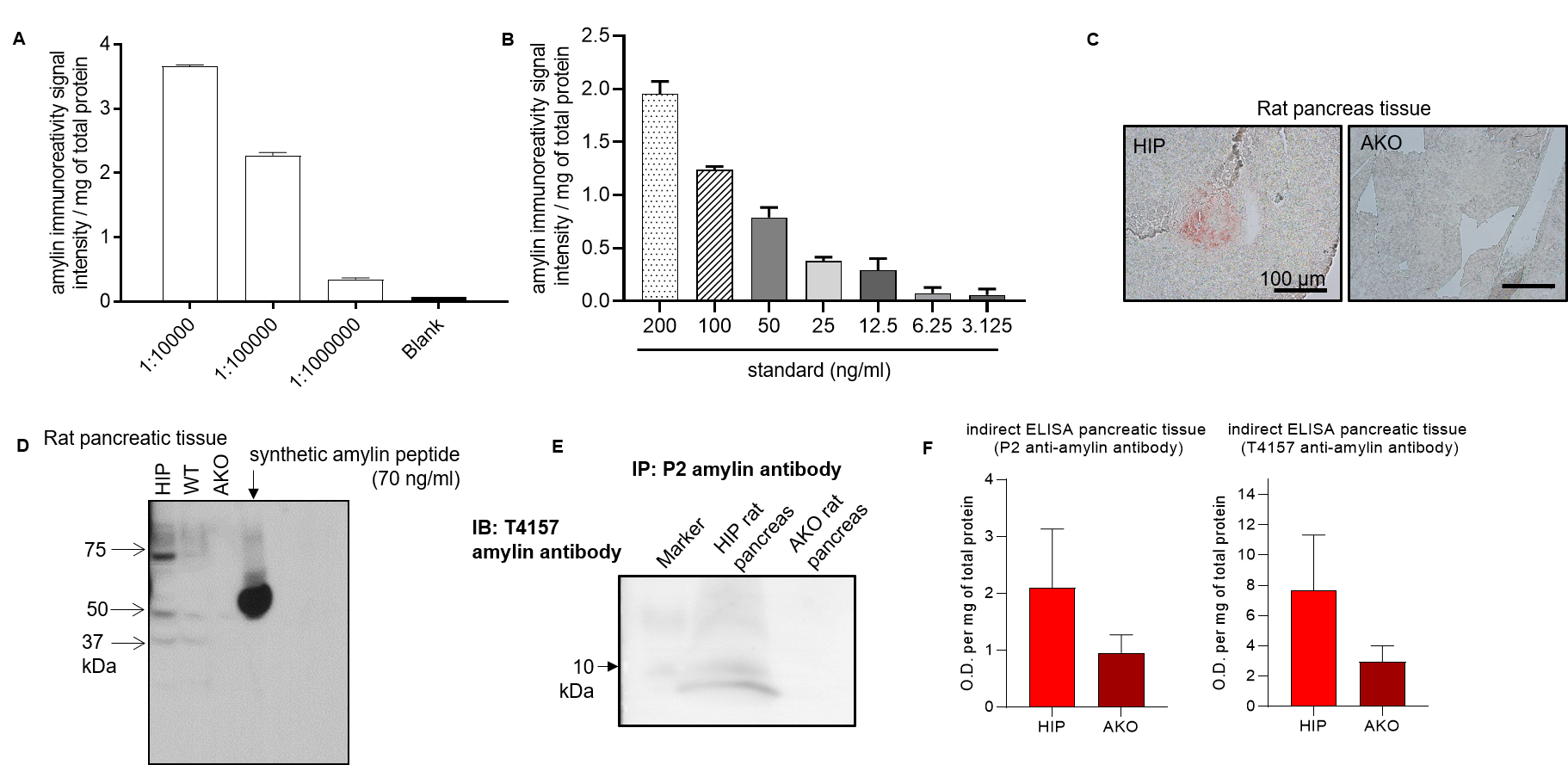


**Supplemental Figure S4. Characterization of P2 anti-amylin antibody.** *A,* P2 amylin antibody titer evaluation by indirect ELISA showing amylin immuno-reactivity signals (normalized to total protein) measured with different dilutions (1:10000 to 1:1000000) of P2 amylin detection antibody. *B,* Amylin indirect ELISA showing amylin immuno-reactivity signals (normalized to total protein) measured in different dilutions (200 ng/ml to 3.125 ng/ml) of amylin using the P2 anti-amylin antibody as a detection antibody. *C,* Representative images of immunohistochemistry (IHC) analysis of pancreas sections from HIP (expresses human amylin in pancreas) and AKO (amylin knock out) rats stained with the P2 anti-amylin antibody showing amylin deposition in presumably a pancreatic islet, in a HIP rat. *D,* Western blot analysis of amylin in pancreatic tissue homogenates from age-matched HIP, WT, and AKO rats along with synthetic human amylin peptide (70 ng/ml). *E,* Immunoprecipitation of amylin with P2 anti-amylin antibody and Western blot analysis of amylin with the T4157 anti-amylin antibody in pancreatic tissue homogenates from HIP and AKO rats. *F,* Amylin indirect ELISA showing amylin immuno-reactivity signal intensities (normalized to total protein) measured in pancreatic tissue homogenates from age matched HIP and AKO rats (*n=3* males/group) using P2 and T4157 anti-amylin antibodies. Data are means ± S.D. in *(F)*; Scale bars 100 μm in *(C).*
