# Supplementary material for: Rapid, scalable assay of amylin-β amyloid co-aggregation in brain tissue and blood
Source: J Biol Chem. 2023 Apr 6;299(5):104682. doi: 10.1016/j.jbc.2023.104682 (PMC10192925; doi:10.1016/j.jbc.2023.104682)
Supplement: Supporting information [file mmc2.docx]

**Amylin-Aβ Sandwich ELISA**

**Day 1**

1. Dilute mouse anti-human-Aβ [1:400 dilution in Bicarbonate buffer (0.028 M Na_2_CO_3_, 0.071 M NaHCO_3_, pH 9.6); clone 6E10, 803002, Biolegend] antibody and coat the wells for standard (first two columns). Dilute rabbit anti-amylin P2 antibody (1:400 dilution in Bicarbonate buffer pH = 9.8, 2 mg/ml stock) and coat the wells for samples with 100 ul volume per well. Seal the plate and incubate overnight at 4 ֯C without shaking.

**Day 2**

1. On the day of assay, bring all reagents to room temperature (RT) prior to use.
2. Prepare the standard and Samples. For standard preparation, resuspend the Aβ_40_/Aβ_42_ peptide in 1ml of DMSO to make 1 mg/ml solution. Prepare the standard by serial dilution in assay diluent (421203, Biolegend). Standards (ng/ml) 10, 5, 2.5, 1.25, 0.625, 0.3125, 0.1562 and blank with assay diluent only.
3. Decant the antibody and remove the residual amount from all wells by inverting the plate and tapping it onto absorbent towels.
4. Wash the plate with 300 μl of washing buffer PBST (PBS with 0.05% Tween-20). Decant and tap after each wash to remove residual buffer.
5. Add 300 μl of Biolegend Blocker (Dilute as 5X in PBS) in each well and incubate at room temperature for 1 hrs without shaking.
6. Decant the blocker and remove the residual amount from all wells by inverting the plate and tapping it onto absorbent towels.
7. Wash the plate with 300 μl PBST (PBS with 0.05% Tween-20) 1-2 times. Decant and tap after each wash to remove residual buffer.
8. Add 100ul of Aβ_40_/Aβ_42_ standards to each well coated with Aβ 6E10 antibody only. And add 100 μl /(50 μl + 50 μl PBS) samples in the rest of the wells coated with amylin P2 antibody. Add 100 ul of PBS/assay diluent as a blank for standard (in Aβ antibody coated well) and sample (for amylin P2 antibody coated well).
9. Seal the plate and put it overnight at 4 ֯C without shaking.

**Day 3**

1. Prepare the detection antibody mouse anti-human-total-Aβ (1:400, clone-4G8, 800720 – Biolegend) or mouse anti-human-Aβ_42_ (1:400, 805507, Biolegend) or mouse anti-human-Aβ_40_ (1:400, 805407, Biolegend) conjugated HRP detection by diluting in assay diluent (1:400).
2. Decant the standard/samples and remove the residual amount from all wells by inverting the plate and tapping it onto absorbent towels.
3. Wash the plate with 300 μl PBST (PBS with 0.05% Tween-20) **three** times. Decant and tap after each wash to remove residual buffer.
4. Add 100 μl/well detection antibody Amyloid-β-HRP conjugate and incubate at room temperature for 1 hr. without shaking.
5. Decant the antibody and remove the residual amount from all wells by inverting the plate and tapping it onto absorbent towels.
6. Wash the plate with 300 μl PBST (PBS with 0.05% Tween-20) 3-4 times. Decant and tap after each wash to remove residual buffer.
7. Add 100 μl substrate solution (TMB - Thawed at room temperature, 34028, Thermo Scientific). Incubate ~30 minutes until you see the signal in standard and samples.
8. Add 50 μl of stop solution (N600, Thermo Scientific) to each well in the same order as substrate was added. Read at 450 nm in a spectrophotometer.
9. Calculate the relative optical density values after subtracting the optical density of standard blank and sample blank from the optical density of standards and samples respectively.
10. Calculate amylin-Aβ concentrations by generating an appropriate standard curve plotting the relative O.D. 450. of each standard (Y) vs. the respective concentration of the standard solution (X). The amylin-Aβ concentrations of the samples can be interpolated from the standard curve.
